# The influence of prior experience and expected timing on vibrotactile discrimination

**DOI:** 10.3389/fnins.2013.00255

**Published:** 2013-12-24

**Authors:** Muhsin Karim, Justin A. Harris, Angela Langdon, Michael Breakspear

**Affiliations:** ^1^School of Psychiatry, The University of New South WalesSydney, NSW, Australia; ^2^The Black Dog InstituteSydney, NSW, Australia; ^3^School of Psychology, The University of SydneySydney, NSW, Australia; ^4^Systems Neuroscience Group, QIMR Berghofer Medical Research InstituteBrisbane, QLD, Australia; ^5^Royal Brisbane and Women's HospitalBrisbane, QLD, Australia

**Keywords:** bias, decision making, perceptual, prior, timing, vibrotactile

## Abstract

Vibrotactile discrimination tasks involve perceptual judgements on stimulus pairs separated by a brief interstimulus interval (ISI). Despite their apparent simplicity, decision making during these tasks is biased by prior experience in a manner that is not well understood. A striking example is when participants perform well on trials where the first stimulus is closer to the mean of the stimulus-set than the second stimulus, and perform comparatively poorly when the first stimulus is further from the stimulus mean. This “time-order effect” suggests that participants implicitly encode the mean of the stimulus-set and use this internal standard to bias decisions on any given trial. For relatively short ISIs, the magnitude of the time-order effect typically increases with the distance of the first stimulus from the global mean. Working from the premise that the time-order effect reflects the loss of precision in working memory representations, we predicted that the influence of the time-order effect, and this superimposed “distance” effect, would monotonically increase for trials with longer ISIs. However, by varying the ISI across four intervals (300, 600, 1200, and 2400 ms) we instead found a complex, non-linear dependence of the time-order effect on both the ISI and the distance, with the time-order effect being paradoxically stronger at short ISIs. We also found that this relationship depended strongly on participants' prior experience of the ISI (from previous task titration). The time-order effect not only depends on participants' expectations concerning the distribution of stimuli, but also on the expected timing of the trials.

## Introduction

Vibrotactile discrimination tasks have been used to examine the behavioral and neural responses involved in perceptual decision making. A fundamental process in perceptual decision making involves the integration of prior task information with current perceptual beliefs. Earlier presentations of vibrotactile stimuli during a task (Sinclair and Burton, 1996; Preuschhof et al., 2010), or training/titration prior to a vibrotactile discrimination study (Karim et al., 2012), have been shown to establish the conditions for biased decision making to occur. Specifically, decisions in these tasks are influenced by an implicit mechanism for evaluating current sensory information in the context of past information garnered from the task. This decision bias is known as the “time-order effect.” After performing a sequential discrimination task for a period of time, participants demonstrably learn an internal standard representing the average of the stimulus-set (the “global mean” representation) (Preuschhof et al., 2010). “Preferred” and “nonpreferred” time-order trials can be categorized according to the orientation of first stimulus magnitude (Stim1) with respect to the global mean and the second stimulus magnitude (Stim2). When Stim1 lies between Stim2 and the global mean, the trial follows the “preferred′' sequence as reflected in faster and more accurate responses. Depending on the stimulus-set used, all other trial sequences are “nonpreferred.” While Stim1 is held in memory during the inter-stimulus interval (ISI) prior to Stim2 onset, the representation of Stim1 effectively “drifts” toward the global mean (Preuschhof et al., 2010). Preferred trials are those in which the Stim1 representation drifts *away* from the Stim2 representation, causing the two stimuli to be perceived as more distinct. Nonpreferred trials are those in which the Stim1 representation drifts *toward* the Stim2 representation, causing the two stimuli to be perceived as less distinct, thus, resulting in decreased accuracy and slower responses (Sinclair and Burton, 1996; Preuschhof et al., 2010).

Knowledge of, and research into the time-order effect dates from the seminal work of Hellström [see (Hellstrom, 1985; Hellström, 2003)] and earlier researchers [see (Needham, 1935; Woodrow, 1935; Michels and Helson, 1954)]. The time-order effect has been explicitly examined in a number of sequential discrimination studies, conducted across different modalities (Masin and Agostini, 1990; Hellström and Rammsayer, 2004; Preuschhof et al., 2010; Alcala-Quintana and Garci, 2011). Its effects are also likely present in a large number of studies where it was not explicitly modeled, contributing to unexplained variance in these data. Various drivers of the time-order effect have been proposed, including response bias, sensation weighting and the aforementioned loss of perceptual precision during memory retention (Hellström, 1979; Masin, 1995; Preuschhof et al., 2009). A number of additional factors may also influence the time-order effect, including desensitization to the magnitude of stimuli due to short ISIs or inter-trial interval durations (Alcala-Quintana and Garci, 2011). If one conceptually frames the time-order effect as an effective drift of the first stimulus toward the global mean, this drift may be faster, and hence the time-order effect stronger, the further the first stimulus (Stim1) is from the global mean. In a recent study, we observed more accurate performance for nonpreferred time-order trials when the “distance” (the difference) between Stim1 and the average of stimulus frequencies was smaller (classified as “closer”), compared to trials in which distance was greater (classified as “further”), consistent with such an interpretation. While these observations support the notion of a perceptual representation drift during the retention period, other interpretations are possible such as forming a perceptual representation that gives weight to both the magnitude of the first stimulus as well as the task stimulus-set “global mean.” This is known as “sensory weighting” (Michels and Helson, 1954; Karim et al., 2012).

The nature of how the time-order effect varies across short intervals of time is incompletely understood. In our previous study, all trials were presented with a fixed ISI of 600 ms, thus, did not explicitly address the temporal profile of the time-order effect with ISI. However, Sinclair and Burton (1996) observed that the magnitude of the time-order effect varied across trials with ISIs ranging from 0.5 up to 30 s (Sinclair and Burton, 1996). In particular, they found the accuracy of nonpreferred time-order trials to decrease relative to preferred trials as a function of increasing ISI. A more thorough investigation of the influence of ISI on the magnitude of the time-order effect is central to understanding its influence on perceptual decision making and necessary to disambiguate between competing interpretations of its origin. Considering this, the objective of the current study was to explicitly study this factor in a paired forced choice vibrotactile discrimination task across a range of ISIs (300, 600, 1200, and 2400 ms). In doing so, our aims were to explore three facets of perceptual decision making in vibrotactile discrimination. First, we examined how the time-order effect changes as a function of ISI. We conjectured that the time-order effect would increase at longer ISIs since the first stimulus representation was expected to “drift” further toward the global mean. Second, we investigated how the time-order effect is influenced by the “distance” of Stim1 to the global mean, and how this varies with increasing ISI. We expected the shift in representation of Stim1 during memory retention to be greater if it is further from the global mean. Third, we sought to determine whether these two factors, time-order and distance, interact. The presence of an interaction would suggest a more complex process than the passive perceptual “drift” that has been postulated.

## Materials and methods

### Ethics statement

Participants gave written informed consent and the study was approved by the University of New South Wales Human Research Ethics Committee. Participants were paid for their participation in the study. All participants were right-handed. Exclusion criteria were the history of a psychiatric disorder, neurological disorder, or any drug or alcohol dependence by self-report.

### Materials

Vibrations were delivered perpendicular to the skin surface of the right index finger via a 2 mm diameter Perspex probe attached to the shaft of a mechanical vibrator (Gearing and Watson, model GWV4, UK). The vibrator was mounted on an isolated rigid trunnion (Gearing and Watson, T4). The probe tip projected through a 6 mm diameter hole in a rigid Perspex plate (300 mm^2^) suspended from the rigid trunnion. The plate was positioned parallel to the skin surface and acted as a guard to limit the spread of surface waves across the skin. The probe and the rigid surround were separated by a gap of 2 mm. Vibrations were generated by a computer equipped with Matlab (version 2007b, Mathworks), using the Psychophysics Toolbox extensions (Brainard, 1997; Kleiner et al., 2007) and a National Instruments card (USB-6259, National Instruments), passed to a linear power amplifier (Gearing and Watson, PA30) and then delivered to the mechanical vibrator.

### Participants

Sixteen participants completed the vibrotactile discrimination task. The average age was 25.1 years (standard deviation: 4.3, range: 18–34). Nine of the participants were male.

### Detection threshold procedure

**Figure 1:**
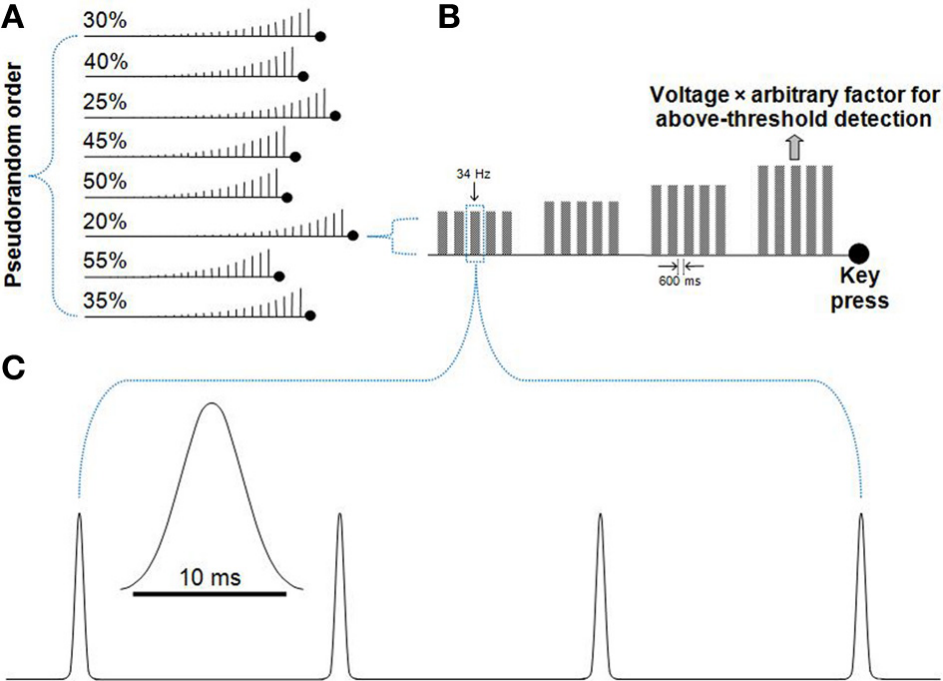
**Detection threshold procedure**. Participants were presented with trains of Gaussian deflections that gradually increased in set step-sizes until the movement of the probe was detected. **(A)** A pseudorandom order of runs with voltage increase step-sizes of 20–55% was presented. For each run, the participant was instructed to press a keyboard key when they perceived the movement of the vibrotactile probe. Accuracy rather than speed was emphasized. **(B)** A series of Gaussian deflections comprised a 34 Hz vibration played in a train of five, separated by 600 ms. If no button was pressed, the next train would step-up in voltage. After the key was pressed, the driving voltage of the probe was recorded. The average driving voltage from the eight runs shown in **(A)** was used to derive each participant's amplitude for the subsequent titration procedure and main task, where a large arbitrary factor (50 for the titration procedure, 30 for the main task) was used to multiply each participant's voltage value to well-above detection threshold amplitude. **(C)** The 34 Hz Gaussian deflections played for 512 ms (17 Gaussian deflections, with the diagram showing four deflections). Each deflection had the form of a truncated Gaussian of 10 ms duration (Lak et al., 2008).

The presented vibrotactile stimuli consisted of a sequence of Gaussian-shaped probe deflections presented at a fixed rate for 512 ms. The width of each Gaussian deflection was sigma equal to 1 and tails were clipped to zero at a maximum width of 10 ms (Figure 1).

A detection threshold procedure was used to determine a subject-specific driving voltage for the amplitude of the vibrotactile stimulus to be used in the task. Each participant was presented with a sequence of stimulus blocks of fixed frequency and step-wise increasing amplitude. Each fixed-amplitude block consisted of five vibrotactile trains of 512 ms, each separated by 600 ms. During a run, participants indicated when they perceived the movement of the probe. Eight runs were conducted, each with a fixed amplitude step-increase between 20 and 55%, and presented in pseudorandom order. The average driving voltage at which each participant detected probe movement across these eight runs was then multiplied by a large arbitrary factor to ensure the stimulus amplitude was well above the detection threshold for each participant. An arbitrary factor of 50 was used for the titration procedure (Figure 1). A lower arbitrary factor of 30 was used for the main task. Even though all study participants were presented with a stimulus-set of more intense vibrations during the titration procedure, the vibrations for the subsequent main task remained well-above detection threshold (as evidenced by high accuracies for the main task).

### Titration procedure

The aim of the titration procedure was to derive an equivalent frequency difference for each participant that matched their performance accuracy, minimizing this inter-subject variability. To address potential response bias, half of the participants were assigned the question “Is the 2nd vibration faster?” whilst the other half answered the question “Is the 2nd vibration slower?” Participants were instructed to answer as quickly and accurately as possible following the onset of the second stimulus.

Participants placed their right index finger on the vibrotactile probe and placed two fingers of their left hand on the left and right arrow keyboard response keys for the titration procedure and main task described below. One key was assigned “Yes” as a response with the other assigned “No.” The positioning (left and right) of the two responses was counterbalanced across participants.

An adaptive staircase procedure was used based on the up-down transformed rule method (Zwislocki and Relkin, 2001; Karim et al., 2012). Stimulus vibrations were 512 ms in duration separated by an ISI of 600 ms. The presentation order of base (34 Hz) and comparison (varied with performance) vibrations was pseudorandomly varied. An “easy” and “hard” staircase was used and the two staircases were randomly intermixed to limit a learning effect from consecutive easy or consecutive hard trials. Starting with a 5 Hz frequency difference, a 10% frequency difference increase (step-up) occurred for each incorrect response. For the easy staircase, a decrease (step-down) occurred after six non-consecutive correct responses. For the hard staircase, a step-down occurred after two non-consecutive correct responses. Each staircase ended when a sliding window of 20 trials reached the proportion correct targets set at 90% (easy, after a minimum of 40 trials) and 65% (hard, after a minimum of 20 trials). The average of the unique step-up and step-down points within the easy staircase window served as the participants' titrated frequency difference value for the main task. Any participant exceeding this value after two attempts of the titration procedure was excluded from the study. One participant was excluded from the first experiment of this study (“Experiment 1”) and two participants were excluded from the second experiment (“Experiment 2”).

### Main task

**Figure 2:**
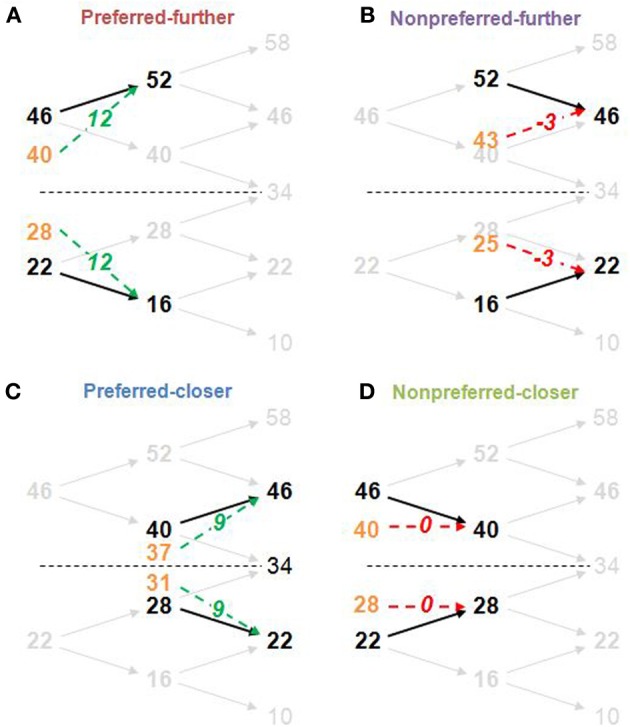
**Structure of derived stimulus-set for the main task and the four time-order/distance trial-types**. The frequency difference between stimuli pairs was determined by the titration procedure (in this example, the frequency difference is 6 Hz). Example trial-types **(A**, Preferred-further; **B**, Nonpreferred-further; **C**, Preferred-closer; **D**, Nonpreferred-closer**)** are shown by thick black frequencies and arrows against the faded stimulus-set used in the task. Orange values show the midpoint between the first stimulus (Stim1) and the global mean (34 Hz) and represents the “perceived drift” value of the first stimulus (perceived Stim1). The “perceived difference” due to this drift is represented by green dotted arrows for preferred time-order trials (more distinct), and by red dotted arrows for nonpreferred time-order trials (less distinct) with the perceived difference (second stimulus frequency minus the perceived Stim1) shown in italics. For example, for the nonpreferred-further trials, the perceived difference is “−3,” indicating that the first stimuli drifted to a position below that of the second stimuli. The “closer” trial-types are defined by a shorter distance between the first stimuli to the global mean compared to the “further” counterparts. The study structure design imposed an absolute maximum titrated frequency difference of 8 Hz. Any participant exceeding this value after two attempts of the titration procedure was excluded from the study (see Titration procedure in Materials and Methods). Frequencies above and below the stimulus-set range (10 and 58 Hz, and an additional frequency of 34 Hz) ensured that participants compared the two vibrations for all other trials rather than making a categorical judgement about the frequency of the second vibration independently of the first (Hernandez et al., 1997).

A stimulus-set structure was designed in order to create counterbalanced preferred and nonpreferred time-order trials above and below the global stimulus mean, as well as counterbalanced “closer” and “further” trials. There were 8 trial-types equally spaced around 34 Hz representing all possible trial-types in this 2 (time-order: preferred/nonpreferrd) × 2 (distance: closer/further) × 4 (ISI: 300, 600, 1200, 2400 ms) factorial structure. Each participant's titrated frequency difference value was used to determine the stimulus magnitudes for each individual. In order to ensure that participants compared the two vibrations for all analyzed trials rather than making a categorical judgement based purely on the frequency of the second vibration (Hernandez et al., 1997), four additional frequencies were added to the task set but not included in the subsequent analysis (see Figure 2 for an example).

The main task was separated into six sessions, each taking approximately 6 min to complete. For each session, all vibration pairs were presented once for each of the four ISIs (*N* = 6 for each pair across the main task). In total, 288 trials, including the pairs with frequencies added to limit categorical judgements were presented to each participant (see Figure 2 for details). Pairs were presented pseudorandomly in each session. Responses for each trial were made in the same Yes/No manner as described for the titration procedure for each participant's respective question (“Is the 2nd vibration faster?” or “Is the 2nd vibration slower?”).

The sensory-weighting approach is a simple heuristic that has been proposed to describe the time-order effect (Michels and Helson, 1954; Hellström, 1979; Hellström and Rammsayer, 2004). For illustrative purposes, we relate this to our experimental design (Karim et al., 2012). At its simplest, the sensory weighting approach states that the “perceived Stim1” is obtained by a 50:50 average of Stim1 and the global mean magnitudes (orange frequencies at the midpoint between Stim1 and the global mean in Figure 2). The “perceived difference” is Stim2 minus the perceived Stim1. Examples for representative preferred and nonpreferred trial types are given in Figure 2.

### Data analysis

Participants' performance was assessed with two dependent variables—accuracy calculated as d-prime (d′), and the speed measure of response time (RT). d′ is a measure of sensitivity that takes participants' response bias into account using the participant's hit and false alarm rates. Consider a participant responding to the question “Is the 2nd vibration faster?” A hit occurs when the second vibration is indeed faster (target is present) and the participant correctly responds “Yes.” A false alarm occurs when the target is absent (“distracter”—second vibration is slower) yet the participant incorrectly answered “Yes.” This logic applies for the reversed question “Is the 2nd vibration slower?”—a hit is when the second vibration is slower and the participant answers “Yes” and a false alarm occurs when the second vibration is faster and the participant answers “Yes.” d′ is calculated as follows:

$${\text{d}}^{\prime }=\left[z(H)-z(F)\right]/\surd 2,$$

where (*H)* is the hit rate and (*F*) is the false alarm rate, both z-transformed. As this was a two-alternative forced-choice task design, d′ values were adjusted downward by a factor of √2. A value of 0.5 was added to all of the data cells (hits, misses, false alarms, and correct rejections) to account for cases of perfect accuracy which would otherwise result in an infinite d′ (Macmillan and Creelman, 2005). A d′ value of zero indicates chance performance.

Analyses were conducted using PASW 18.0 Statistical Package (SPSS, Inc., Chicago, Illinois). Repeated measures analyses of variance (ANOVA) were used to compare within-subject differences in behavioral performance across the different trial-types of the main task; the influence of time-order (preferred, nonpreferred), distance (closer, further), and ISI (300, 600, 1200, 2400 ms) on behavioral data. Because ISI varied across four intervals, we examined linear, quadratic, and cubic effects for this factor. For clarity of presentation, most statistics are presented in table form, directing the reader to these in the text using curly brackets {}.

## Results

### Titration task

Only the frequency difference corresponding to the easy staircase (not the hard staircase) was employed the main experiment. The average of each participant's frequency difference derived from the titration procedure was 5.86 Hz (standard deviation: 1.70 Hz, range: 2.93–7.92 Hz).

### Main experiment

**Figure 3:**
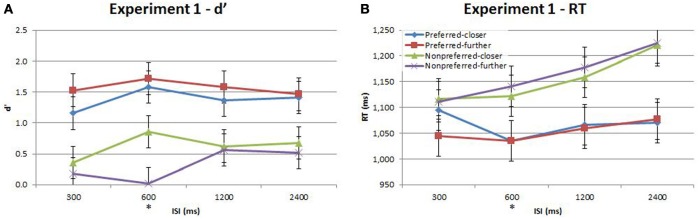
**Accuracy (d′) and response time (RT) for the time-order, distance and ISI analysis for Experiment 1**. Each plot shows the behavioral performance across the four ISIs of four time-order/distance trial-types: preferred-closer (blue), preferred-further (red), nonpreferred-closer (green) and nonpreferred-further (purple). **(A)** Experiment 1—accuracy (d′). **(B)** Experiment 1—response time (RT). The star on the y-axis indicates that Experiment 1 participants were only presented with an ISI of 600 ms during the titration procedure. Vertical bars represent within-subject SEM for the distance factor.

**Figure 4:**
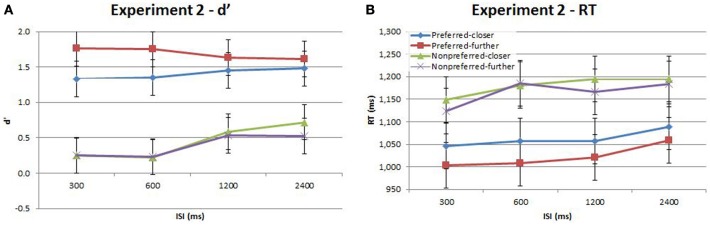
**Accuracy (d′) and response time (RT) for the time-order, distance and ISI analysis for Experiment 2**. Each plot shows the behavioral performance across the four ISIs of four time-order/distance trial-types: preferred-closer (blue), preferred-further (red), nonpreferred-closer (green) and nonpreferred-further (purple). **(A)** Experiment 2—accuracy (d′). **(B)** Experiment 2—response time (RT). Vertical bars represent within-subject SEM for the factor of distance.

Performance during the main experiment is illustrated in Figures 3, 4, and summarized separately for accuracy (d′) and speed (response time; RT) in Table 1. Inspection of these data immediately suggest a very strong time-order effect, manifest as a marked difference in both performance measures for the preferred to the nonpreferred trials. Also of note is the marked divergence in accuracy for nonpreferred trials at 600 ms as a function of the distance factor. Quantitative analysis showed that there was indeed a statistically significant main effect on accuracy for the time-order factor (preferred/nonpreferred)^{1a}^ but not for distance (closer/further)^{1b}^. As expected, accuracy was greater for preferred time-order trials compared to nonpreferred time-order trials (Figure 3A). There was a significant main effect of ISI, in particular, there were both statistically significant linear and quadratic trends in the increase in accuracy as ISI duration increased^{1c}^. The quadratic effect reflects the divergence of d′ in the nonpreferred but not the preferred trial-types (Figure 3A). There was a significant interaction between time-order and distance^{1d}^ and between distance and ISI^{1e}^ on accuracy. These interactions were driven by the greater accuracy of nonpreferred-closer trials at 600 ms compared to chance performance at the same ISI for nonpreferred-further trials (Figure 3A).

**Table 1:** **Experiment 1 accuracy (d′) and response time (RT) statistics of the time-order × distance × ISI repeated measures ANOVA**.

**Factor**	***F*-statistic and *p*-value**	**ISI trend**			**Text ref**.
		**Linear**	**Quadratic**	**Cubic**	
**d′**					
Time-order	*F*_(1, 15)_ = 56.069	*NA*	*NA*	*NA*	{1a}
	*p* < 0.0001^*^				
Distance	*F*_(1, 15)_ = 0.385	*NA*	*NA*	*NA*	{1b}
	*p* = 0.5441				
ISI	*F*_(3, 45)_ = 8.204	*F*_(1, 15)_ = 9.540	*F*_(1, 15)_ = 9.136	*F*_(1, 15)_ = 3.212	{1c}
	*p* = 0.0002^*^	*p* = 0.0075^*^	*p* = 0.0086^*^	*p* = 0.0933	
Time-order × distance	*F*_(1, 15)_ = 15.676	*NA*	*NA*	*NA*	{1d}
	*p* = 0.0013^*^				
Time-order × ISI	*F*_(3, 45)_ = 1.358	*NS*	*NS*	*NS*	
	*p* = 0.2678				
Distance × ISI	*F*_(3, 45)_ = 3.221	*F*_(1, 15)_ = 0.001	*F*_(1, 15)_ = 2.042	*F*_(1, 15)_ = 10.634	{1e}
	*p* = 0.0314^*^	*p* = 0.9780	*p* = 0.1736	*p* = 0.0053^*^	
Time-order × distance × ISI	*F*_(3, 45)_ = 2.615	*NS*	*NS*	*NS*	
	*p* = 0.0627				
**RT**					
Time-order	*F*_(1, 15)_ = 42.165	*NA*	*NA*	*NA*	{1f}
	*p* < 0.0001^*^				
Distance	*F*_(1, 15)_ = 0.017	*NA*	*NA*	*NA*	
	*p* = 0.8993				
ISI	*F*_(3, 45)_ = 6.050	*F*_(1, 15)_ = 7.893	*F*_(1, 15)_ = 4.284	*F*_(1, 15)_ = 1.261	{1g}
	*p* = 0.0015^*^	*p* = 0.0132^*^	*p* = 0.0562	*p* = 0.2792	
Time-order × distance	*F*_(1, 15)_ = 0.719	*NA*	*NA*	*NA*	
	*p* = 0.4098				
Time-order × ISI	*F*_(3, 45)_ = 4.523	*F*_(1, 15)_ = 12.742	*F*_(1, 15)_ = 0.029	*F*_(1, 15)_ = 1.124	{1h}
	*p* = 0.0074^*^	*p* = 0.0028^*^	*p* = 0.8672	*p* = 0.3057	
Distance × ISI	*F*_(3, 45)_ = 0.690	*NS*	*NS*	*NS*	
	*p* = 0.5629				
Time-order × distance × ISI	*F*_(3, 45)_ = 0.235	*NS*	*NS*	*NS*	
	*p* = 0.8714				

*Statistics for ISI trends (linear, quadratic, cubic) are shown. ISI trend statistics have not been shown when the interaction between ISI and another factor (or factors) was not significant. NA—not applicable. NS—not shown*.

* *indicates p-value < 0.05*.

There was also a significant difference across time-order trials^{1f}^ for RT. Interestingly, with an increase in ISI, the RT increased for the nonpreferred trials but not for the preferred time-order trials (Figure 3B). There was a significant effect across ISIs^{1g}^ and a significant interaction between time-order and ISI^{1h}^. The significant linear trend^{1h}^ appears to be driven by the increase in RT on the nonpreferred trials with an increase in ISI (Figure 3B).

A stand-out feature of these data is the marked divergence in accuracy between closer and further nonpreferred trials at 600 ms. Since this corresponds exactly with the ISI employed in the titration procedure, it is natural to ask whether prior experience on this specific trial timing indeed underlies this effect. In order to test this hypothesis, we conducted a second experiment in which a new cohort of participants was exposed to all four ISIs during the titration procedure, prior to the main task. We hereafter refer to the Results presented above as “Experiment 1” and to this new task as “Experiment 2.”

The titration procedure for Experiment 2 contained four intermixed staircases for each ISI of 300, 600, 1200, and 2400 ms. However, the termination criterion was derived from the performance only on the 600 ms staircase. The staircase ended when a sliding window of 20 trials reached the proportion correct target of 85% (for Experiment 1, the target was 90%). Because of time and fatigue considerations, the task was allowed to terminate after the participant had first performed a minimum of 20 trials (for Experiment 1, it was 40 trials). Trials from the four staircases were presented to the participants in a pseudorandom fashion in a set of 8 trials, such that each staircase was represented equally (two ISI trials each). After the set of eight, a new pseudorandom set was presented. This ensured that the participant received an approximately equal number of trials from each ISI staircase until the criteria was reached.

The average of each participant's frequency difference derived from this titration procedure was 5.76 Hz (standard deviation: 1.11 Hz, range: 4.29–7.89 Hz). There was no significant difference between the titrated frequency difference values between Experiment 1 and Experiment 2 participants despite the different proportion correct targets across the two separate titration procedures [*t*_(25.794)_ = −0.1929, *p* = 0.8486 [2-tailed], corrected for unequal variance].

Participants in Experiment 2 then undertook the same main task as in Experiment 1. The accuracy and response times are shown in Figure 4 and Table 2. As in Experiment 1, there was a strong and significant effect of the time-order effect on accuracy^{2a}^. There was a significant linear, but not quadratic effect for ISI^{2b}^. The most striking finding in Experiment 2 is that the marked divergence between closer and further trials at 600 ms that was present in Experiment 1 for nonpreferred trials is *not* present in Experiment 2. This is reflected in changes in the observed interaction effects. For example, there is now a significant interaction between time-order and distance^{2c}^. For the preferred time-order trials, the difference in accuracy between the closer and further distance trials is greater compared to nonpreferred trials (Figure 4A). As before, there was a significant linear interaction between time-order and ISI^{2d}^. There appears to be an increase in accuracy at greater ISIs (1200 and 2400 ms) amongst the nonpreferred time-order trials (Figure 4A). As for Experiment 1, there was a significant effect across time-order trials for RT^{2e}^, and a significant linear trend across ISIs^{2f}^. Figure 4B clearly indicates that with an increase in the ISI between stimuli, the participants' RT increases, as expected.

**Table 2:** **Experiment 2 accuracy (d′) and response time (RT) statistics of the time-order × distance × ISI repeated measures ANOVA**.

**Factor**	***F*-statistic and *p*-value**	**ISI trend**			**Text ref**.
		**Linear**	**Quadratic**	**Cubic**	
**d′**					
Time-order	*F*_(1, 15)_ = 50.138	*NA*	*NA*	*NA*	{2a}
	*p* < 0.0001^*^				
Distance	*F*_(1, 15)_ = 1.643	*NA*	*NA*	*NA*	
	*p* = 0.2194				
ISI	*F*_(3, 45)_ = 3.072	*F*_(1, 15)_ = 5.229	*F*_(1, 15)_ = 0.229	*F*_(1, 15)_ = 1.404	{2b}
	*p* = 0.0371^*^	*p* = 0.0372^*^	*p* = 0.6394	*p* = 0.2545	
Time-order × distance	*F*_(1, 15)_ = 11.266	*NA*	*NA*	*NA*	{2c}
	*p* = 0.0043^*^				
Time-order × ISI	*F*_(3, 45)_ = 3.527	*F*_(1, 15)_ = 6.679	*F*_(1, 15)_ = 0.234	*F*_(1, 15)_ = 1.556	{2d}
	*p* = 0.0222^*^	*p* = 0.0207^*^	*p* = 0.6356	*p* = 0.2314	
Distance × ISI	*F*_(3, 45)_ = 1.647	*NS*	*NS*	*NS*	
	*p* = 0.1919				
Time-order × distance × ISI	*F*_(3, 45)_ = 0.212	*NS*	*NS*	*NS*	
	*p* = 0.8877				
**RT**					
Time-order	*F*_(1, 15)_ = 31.484	*NA*	*NA*	*NA*	{2e}
	*p* < 0.0001^*^				
Distance	*F*_(1, 15)_ = 2.367	*NA*	*NA*	*NA*	
	*p* = 0.1448				
ISI	*F*_(3, 45)_ = 5.927	*F*_(1, 15)_ = 15.912	*F*_(1, 15)_ = 0.117	*F*_(1, 15)_ = 1.511	{2f}
	*p* = 0.0017^*^	*p* = 0.0012^*^	*p* = 0.7372	*p* = 0.2379	
Time-order × distance	*F*_(1, 15)_ = 2.549	*NA*	*NA*	*NA*	
	*p* = 0.1312				
Time-order × ISI	*F*_(3, 45)_ = 1.236	*NS*	*NS*	*NS*	
	*p* = 0.3076				
Distance × ISI	*F*_(3, 45)_ = 0.138	*NS*	*NS*	*NS*	
	*p* = 0.9369				
Time-order × distance × ISI	*F*_(3, 45)_ = 0.378	*NS*	*NS*	*NS*	
	*p* = 0.7694				

*Statistics for ISI trends (linear, quadratic, cubic) are shown. ISI trend statistics have not been shown when the interaction between ISI and another factor (or factors) was not significant. NA—not applicable. NS—not shown*.

* *indicates p-value < 0.05*.

When participants make sensory discriminations, their sensitivity is tuned to the relative difference rather than the absolute difference between stimuli—a well-established phenomenon known as Weber's Law (Weber, 1834). Therefore, when our participants discriminate between vibrations that have a fixed difference in frequency, their accuracy is likely to be influenced by the absolute frequencies of those vibrations. To take this effect into consideration, we split trials into two groups, those for which the vibrations were below the global mean (“low”) and those above the global mean (“high”). The influence of stimulus frequency was examined alongside the time-order, distance and ISI factors using proportion correct. Proportion correct rather than d′ was used as the dependent measure for accuracy in this analysis because it was no longer possible to calculate a false alarm rate for both preferred and nonpreferred time-order trials once the trials had been split into those above and those below the global mean frequency.

**Figure 5:**
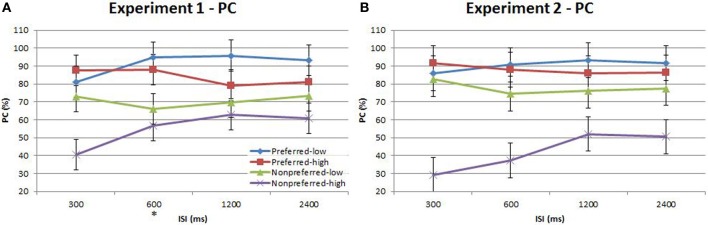
**Proportion correct (PC) of time-order/frequency trial-types across the four ISIs. (A)** Experiment 1. **(B)** Experiment 2. Experiment 2 plot shows below-chance proportion correct toward the nonpreferred-high trials, consistent with the perception of the “opposite direction” (see text for description). The star on the y-axis indicates that Experiment 1 participants were only presented with an ISI of 600 ms during the titration procedure. Vertical bars represent within-subject SEM for the factor of time-order.

There was indeed a significant main effect of the “low/high” frequency factor for both Experiment 1^{3a}^ and Experiment 2^{3b}^ as shown in Table 3. As clearly observed in Figure 5, accuracy was on average lower for trials of high frequency. Using partial eta squared (η^2^) to estimate the variance explained by each factor, the factor time-order was more influential on accuracy than frequency in Experiment 1^{3c, 3a}^. For Experiment 2 however, both time-order (~79%) and frequency (~76%) explained roughly equal amounts of the variance in accuracy^{3d, 3b}^. For Experiment 2, there was a significant interaction between time-order and frequency^{3e}^. Figure 5A shows that accuracy was lower when trials were of the nonpreferred time-order and high frequency, compared to when trials were nonpreferred and low frequency.

**Table 3:** **Accuracy (proportion correct) statistics of the time-order × frequency × ISI repeated measures ANOVA**.

**Factor**	***F*-statistic**	***p*-value**	**η^2^**	**Text ref**.
**EXPERIMENT 1**				
Time-order	*F*_(1, 15)_ = 45.289	*p* < 0.0001^*^	0.7512	{3c}
Frequency	*F*_(1, 15)_ = 8.935	*p* = 0.0092^*^	0.3733	{3a}
ISI	*F*_(3, 45)_ = 7.492	*p* = 0.0004^*^	0.3331	
Time-order × frequency	*F*_(1, 15)_ = 0.601	*p* = 0.4503	0.0385	
Time-order × ISI	*F*_(3, 45)_ = 1.883	*p* = 0.1460	0.1115	
Frequency × ISI	*F*_(3, 45)_ = 0.456	*p* = 0.7140	0.0295	
Time-order × frequency × ISI	*F*_(3, 45)_ = 11.420	*p* < 0.0001^*^	0.4322	
**EXPERIMENT 2**				
Time-order	*F*_(1, 15)_ = 56.268	*p* < 0.0001^*^	0.7895	{3d}
Frequency	*F*_(1, 15)_ = 47.618	*p* = 0.0001^*^	0.7605	{3b}
ISI	*F*_(3, 45)_ = 2.914	*p* = 0.0445^*^	0.1627	
Time-order × frequency	*F*_(1, 15)_ = 8.696	*p* < 0.0100^*^	0.3670	{3e}
Time-order × ISI	*F*_(3, 45)_ = 2.467	*p* = 0.0743	0.1413	
Frequency × ISI	*F*_(3, 45)_ = 2.154	*p* = 0.1067	0.1256	
Time-order × frequency × ISI	*F*_(3, 45)_ = 6.502	*p* = 0.0009^*^	0.3024	

*Eta squared (η^2^) values indicate the relative contribution of influence for each factor by accounting for the respective proportion of variance in the analysis*.

* *indicates p-value < 0.05*.

**Figure 6:**
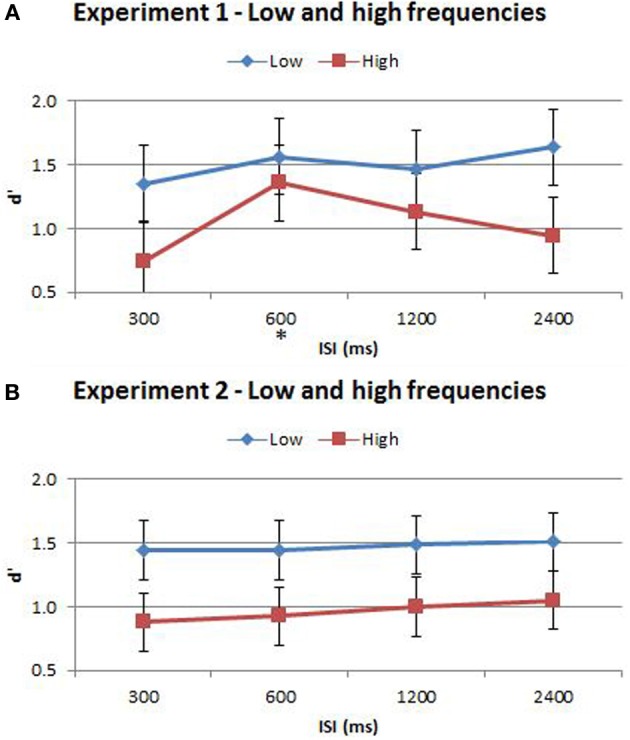
**(A)** Accuracy comparison between low and high frequencies in Experiment 1. **(B)** Accuracy comparison between low and high frequencies in Experiment 2. Vibration pairs below the global mean were classified as “low” whilst those greater than the global mean were classified as “high” frequencies. Accuracy was determined by d′, which served to reduce the effect of potential response bias. The ISI trial-types for low and high frequencies are indicated below each bar. This analysis excluded trial pairs that contained either the maximum and minimum frequencies in the stimulus-set for each participant as these may be too discrete and or beyond the flutter range of vibrations. The star on the y-axis indicates that Experiment 1 participants were only presented with an ISI of 600 ms during the titration procedure. Vertical bars represent within-subject SEM for the frequency factor. Statistical analysis results are displayed below in Table 4.

The split of trials into low/high frequency reveals important additional information regarding the interaction between the time-order effect and ISI. When examining nonpreferred trials without this split (Figures 3, 5) it appears that accuracy measured by d′ is very low (but above chance) at short ISIs, improving for longer ISIs. However, the present analysis suggests that at very short ISIs, performance for high frequency nonpreferred trials (the hardest trial-type in the experiment) is statistically below chance. Performance reverts toward chance levels at longer ISIs. The estimation of d′ in the preceding analyses required combining high and low frequency trials—hence this apparent “below chance” effect at short ISIs in the high frequency-nonpreferred trials was not apparent in these earlier analyses. Unfortunately, because the analysis for Figure 5 uses proportion correct as the dependent variable, we cannot exclude the influence of response bias on these results. It is possible to use d′ as the outcome measure when splitting trials into low/high frequency—but this now requires collapsing across preferred and nonpreferred trials. Analysing performance in this way confirms that accuracy was greater for the low frequencies in both Experiment 1 and Experiment 2 (see Figure 6 and Table 4). Unfortunately we again cannot tease out nonpreferred, high frequency trials. We return to this issue in the Discussion.

**Table 4:** **Statistics for the low and high frequency analysis to examine the impact of Weber's Law by limiting the influence of response bias by using d′**.

**Factor**	***F*-statistic and *p*-value**	**η^2^**
**EXPERIMENT 1**		
Frequency	*F*_(1, 15)_ = 9.641; *p* = 0.0072^*^	0.391
ISI	*F*_(3, 45)_ = 7.693; *p* = 0.0003^*^	0.339
Frequency × ISI	*F*_(1, 15)_ = 3.069; *p* = 0.0373^*^	0.170
**EXPERIMENT 2**		
Frequency	*F*_(1, 15)_ = 0.787; *p* = 0.0005^*^	0.564
ISI	*F*_(3, 45)_ = 0.787; *p* = 0.5072	0.050
Frequency × ISI	*F*_(1, 15)_ = 0.126; *p* = 0.9443	0.008

*There was a significant difference for factors frequency, ISI and the interaction between the two for Experiment 1. There was only a significant effect for frequency for Experiment 1. Eta squared (η^2^) values indicate the relative influence of each factor. For Experiment 1, frequency accounts for ~39% and ISI accounts for ~34% of the variance. For Experiment 2, frequency accounts for ~56% of the variance*.

* *indicates p-value < 0.05*.

## Discussion

We examined the temporal dynamics of perceptual representation by observing performance in a vibrotactile discrimination task. By varying the ISI across four relatively short intervals, we found a complex, nonlinear dependence of the time-order effect on both the ISI and the distance in magnitude between the first stimulus and the global mean. Contrary to our expectations, the time-order effect was strongest at the short ISIs. Furthermore, we found that this relationship depended strongly on participants' prior experience of the ISI range as established from exposure to trials during a titration procedure.

Our first objective was to examine how the strength of the time-order effect varied as a function of ISIs up to 2400 ms. Preuschhof et al. (2009) used ISIs of 100 and 4100 ms, and found a significant increase in the size of the time-order effect with an increase in ISI (Preuschhof et al., 2010). Sinclair and Burton (1996) used ISIs of 0.5, 5, 15, and 30 s—for trials with stimuli around 50 Hz, the accuracy of nonpreferred trials dropped dramatically at ISI of 30 s. We hence hypothesized that the representation of stimulus pairs for preferred trials would diverge from each other to a greater extent over longer ISIs, making the stimuli perceptually more distinct. Conversely, the representation of stimulus pairs for nonpreferred trials would approach each other to a greater degree over longer ISIs, making these stimuli perceptually less distinct. Contrary to these expectations, we observed that the influence of the time-order effect appeared to be stronger at short ISIs (particularly in Experiment 2, Figure 4A). The reduced accuracy for nonpreferred trials may reflect incomplete memory consolidation of Stim1, or desensitization of new stimuli from the immediate presentation of vibrations for the short ISI durations (Alcala-Quintana and Garci, 2011), rather than an increased influence of the time-order effect. Preferred time-order trials are spared these effects as they are more perceptually distinct. Presumably, had we tested longer ISIs we would have found that the time-order effect became stronger with increasing ISI, as reported previously (Sinclair and Burton, 1996; Preuschhof et al., 2010). Our results, in combination with those earlier findings, show that the relationship between ISI and the time-order effect is not monotonic, but rather the time-order effect is stronger for comparisons across very short ISIs (less than 1 s), then becomes weaker for intermediate ISIs (1–2 s) before again become strong across long ISIs.

There was a significant interaction between time-order and ISI for Experiment 2. As Figure 4A indicates, the accuracy toward all nonpreferred time-order trials is just above chance at shorter ISIs (300 and 600 ms have equivalent proportion correct scores of ~56% each) and greater at longer ISIs (1200 and 2400 ms have equivalent proportion correct scores of ~64% each). What may account for just above chance performance toward nonpreferred trials across the different ISIs? Figure 2 summarizes the sensory-weighting heuristic, whereby a perceived stimulus is formed as an average of Stim1 and the stimulus-set global mean. The sensory-weighting approach—giving equal weight to Stim1 and the global mean—suggests that the perceived differences for nonpreferred trials of this task would be zero and −3 Hz (Figure 2B). Chance performance would be expected, or a systematic bias in the “opposite direction” may occur. Using an example of nonpreferred-further trials with Stim1 and Stim2 of 52 and 46 Hz, respectively (Figure 2B), the approach suggests that the perceptual representation of Stim1 would be 43 Hz, and the perceived difference hence −3 Hz. In response to the question “Is the 2nd vibration faster?” the correct answer is “No.” If however, the representation of Stim1 is 43 Hz, then participants would be systematically biased toward a “Yes” response. The sensory-weighting approach could account for the dependence of the time-order effect on ISI by ascribing differently weighted ratios of the global mean and Stim2 according to the ISI. If the ISI is particularly short and Stim1 thus, poorly consolidated at the onset of Stim2, then it is possible that less regard is given to Stim1 compared to the global mean than when the same stimulus frequency is presented during trials with longer ISIs. From previous studies, more weight is given to the global mean when the ISI is very long (Sinclair and Burton, 1996; Preuschhof et al., 2010), potentially because the memory trace of Stim1 begins to decay.

According to Weber's Law, sensitivity in discriminating between two stimuli is inversely proportional to the magnitude of the stimuli. We took account of this additional factor in a separate analysis in which trials were classified as “low” or “high” frequency according to whether the two vibrations were above or below the global mean frequency. Performance was significantly less accurate when discriminating between two high frequency vibrations than two low frequency vibrations, confirming Weber's Law. In Experiment 2, time-order accounted for ~79% and frequency accounted for ~76% of the variance in discrimination accuracy. Thus, time-order and frequency were equally influential for Experiment 2 participants (who received training at all four ISIs). Significant interactions between time-order and frequency were also observed in this experiment. Furthermore, the proportion correct for Experiment 2 participants was below chance at 300 and 600 ms, then at chance for 1200 and 2400 ms (Figure 5B). Thus, the perception of the “opposite direction” at short ISIs, resulting in below-chance accuracy, is only apparent when absolute stimulus frequency is considered in the analysis. This effect is otherwise hidden. Incomplete memory consolidation or stimuli desensitization cannot alone explain the below-chance performance observed for nonpreferred-low trials at the short ISIs of 300 and 600 ms (Figure 4B). Chance performance would be expected if these processes were occurring in isolation. One possible explanation is that in these difficult (high frequency/short ISI) trials, participants struggle to accurately encode, represent and recall Stim1, and hence weigh the global mean disproportionally high, in comparison to trials with longer ISIs. Thus, the time-order effect colludes with poor sensory consolidation to yield a systematic erroneous response. Caution for this analysis must be taken due to the loss in power that follows from splitting the data, and by the potential for response bias as d′ was not used as the accuracy measure. Participants might employ a systematic bias when replacing a poorly encoded Stim1 with a “best guess” estimate. Regardless, the presentation of the low/high data split is important to note because without this split (Figure 3B) this “below-chance” effect is obscured.

Our second aim was to examine how the time-order effect varied with the “distance” of Stim1 to the global mean, and how this effect interacts with ISI. We observed a strong and significant interaction between time-order and distance, driven by the preferred time-order trials. Accuracy was higher for preferred-further trials than for preferred-closer trials in both Experiments (Figures 3A, 5A, respectively). The lack of a closer/further effect in nonpreferred trials is interesting to note. Variation in distance may be negligible for nonpreferred time-order trials since these paired-stimuli are already difficult to perform.

Finally, our third aim was to establish whether there was any interaction between the effects of ISI and distance on the time-order effect. We originally designed our experiment without considering that exposure to a particular ISI during titration would impinge strongly on performance in the main experiment. However, the marked divergence in accuracy between nonpreferred-closer and nonpreferred-further trials specific to ISI 600 ms (Figure 3A) indicated a strong training effect on performance. We ran Experiment 2, ensuring that participants were evenly exposed to all ISIs during titration. The specific effect of divergence between nonpreferred distance trials at ISI 600 ms was not present in Experiment 2. Prior training on a single ISI appears to confer an advantage in accuracy toward nonpreferred-closer trials when presented at the same ISI amongst novel delay periods in a subsequent decision making task. There were however, other differences across the titration procedure between the two independent groups of participants including the titration procedure termination criteria, the average number of trials the two groups received, and the time to complete the titration procedures. Despite the differences in procedures, the striking effect unique to the single titrated ISI in Experiment 1 warrants an explanation. The observed results may be due to an anticipatory bias specific to Experiment 1 (where there was indeed a clearly anticipated timing). Temporal expectations have previously been demonstrated to influence perceptual performance (Nobre et al., 2007) and have been proposed to act via a gain-mediated increase in signal contrast (Rohenkohl et al., 2012). This explanation does not account for why enhancement occurs for nonpreferred-closer trials yet not for nonpreferred-further trials in the current study. In our experiment, there is a strong interaction between these two instantiations of prior experience—between the enhancement of frequency discrimination at expected timings and the influence of the learned distribution of the stimulus distribution. The results indicate that the former exaggerates the distance effect of the latter, so that performance in nonpreferred-further trials is close to chance, whereas performance is enhanced in nonpreferred-closer trials. This raises an interesting question as to how the proposed mechanisms that underlie the enhancement of performance with temporal regularity interact with the bias mechanisms at work in the time-order effect.

Depending on the stimulus presentation order, the term “drift” has been used to describe how the representation of the first stimulus effectively (but perhaps not literally) shifts toward the global mean (Hairston and Nagarajan, 2007; Preuschhof et al., 2010). Decades ago, Woodrow (1935) used the term “gravitated” when describing the time-order effect on duration discrimination tasks (Woodrow, 1935). The notion of a drifting memory trace is useful when conveying how stimuli are being represented by neurons over time. However, there is no direct evidence for such an effect. We have maintained the use of the term “drift” but additionally invoke a very simple sensory-weighting approach that complements the drift description. Hellström's sensation-weighting model permits differential weighting of both stimuli in a trial-pair. It is consistent with accounts of cross-modality integration based on maximum likelihood estimation of sensory representation (Ernst and Banks, 2002) except that information is integrated in time, not across modalities. In addition, it is important to point out that the time-order effect is not necessarily based on a weighted average of Stim1 and the global mean in the sense that this mean is calculated on each or any trial. Rather, it may be that on a trial-by-trial basis, participants use *either* their estimate of Stim1 *or* the global mean as the proxy estimate of Stim1. This is consistent with participants relying on the global mean as a substitute on any trial in which they have failed to represent or remember Stim1. Particularly relevant to this interpretation is the absolute frequency—participants are worse at representing high frequencies than lower ones and are perhaps more likely to rely on the global mean than Stim1 when Stim1 is high. This would drive a stronger time-order effect for high than low frequencies. The other factor to consider in this way is ISI. When the ISI is very short, participants have an increased chance of failing to consolidate Stim1, and in turn increase the probability of their relying on the global mean to perform the discrimination. As the ISI increases, their ability to consolidate Stim1 improves and they rely less on the global mean. But as the ISI becomes much longer, perceptual memory for Stim1 decays and participants again rely more often on the global mean. Importantly, we find that this ISI dependence can be shifted by pre-training. If participants are well practiced at a particular ISI (such as 600 ms) then they are able to efficiently consolidate Stim1 despite the short ISI, and thus, be less reliant on the global mean. Taken this way, these results suggest the time-order effect is a marker for trial difficulty—or at least difficulty in representing Stim1.

### Conflict of interest statement

The authors declare that the research was conducted in the absence of any commercial or financial relationships that could be construed as a potential conflict of interest.
